# Seasonal changes in eicosanoid metabolism in the brown bear

**DOI:** 10.1007/s00114-018-1583-8

**Published:** 2018-09-17

**Authors:** Sylvain Giroud, Alina L. Evans, Isabelle Chery, Fabrice Bertile, Georg Tascher, Justine Bertrand-Michel, Guillemette Gauquelin-Koch, Jon M Arnemo, Jon E. Swenson, Etienne Lefai, Stéphane Blanc, Chantal Simon

**Affiliations:** 1 Department of Integrative Biology and Evolution, Research Institute of Wildlife Ecology, University of Veterinary Medicine, Savoyenstraße 1, 1160 Vienna, Austria; 2 Department of Forestry and Wildlife Management, Inland Norway University of Applied Sciences, NO-2480 Koppang, Norway; 3 University of Strasbourg, IPHC, 23 rue Becquerel, 67087 Strasbourg, France; 4 CNRS, UMR7178, 67087 Strasbourg, France; 5 MetaToul-LIPIDOMIQUE Core Facility, MetaboHUB, Inserm U1048, Toulouse, France; 6 CNES Paris, 2 Place Maurice Quentin, 75039 Cedex 01 Paris, France; 7 Department of Wildlife, Fish and Environmental Studies, Swedish University of Agricultural Sciences, SE-90183 Umeå, Sweden; 8 Faculty of Environmental Sciences and Natural Resource Management, Norwegian University of Life Sciences, PO Box 5003, NO-1432 Ås, Norway; 9 Norwegian Institute for Nature Research, NO-7485 Trondheim, Norway; 10 CARMEN, INSERM U1060 / University of Lyon / INRA U1235, Oullins, France

**Keywords:** Hibernation, Metabolism, Fatty acids, Prostaglandins, Leukotriene, Thromboxane

## Abstract

Polyunsaturated fatty acids (PUFAs) exert several important functions across organ systems. During winter, hibernators divert PUFAs from oxidation, retaining them in their tissues and membranes, to ensure proper body functions at low body temperature. PUFAs are also precursors of eicosanoids with pro- and anti-inflammatory properties. This study investigated seasonal changes in eicosanoid metabolism of free-ranging brown bears (*Ursus arctos*). By using a lipidomic approach, we assessed (1) levels of specific omega-3 and omega-6 fatty acids involved in the eicosanoid cascade and (2) concentrations of eicosanoids in skeletal muscle and blood plasma of winter hibernating and summer active bears. We observed significant seasonal changes in the specific omega-3 and omega-6 precursors. We also found significant seasonal alterations of eicosanoid levels in both tissues. Concentrations of pro-inflammatory eicosanoids, such as thromboxane B2, 5-hydroxyeicosatetraenoic acid (HETE), and 15-HETE and 18-HETE, were significantly lower in muscle and/or plasma of hibernating bears compared to summer-active animals. Further, plasma and muscle levels of 5,6-epoxyeicosatrienoic acid (EET), as well as muscle concentration of 8,9-EET, tended to be lower in bears during winter hibernation vs. summer. We also found lower plasma levels of anti-inflammatory eicosanoids, such as 15dPGJ_2_ and PGE_3_, in bears during winter hibernation. Despite of the limited changes in omega-3 and omega-6 precursors, plasma and muscle concentrations of the products of all pathways decreased significantly, or remained unchanged, independent of their pro- or anti-inflammatory properties. These findings suggest that hibernation in bears is associated with a depressed state of the eicosanoid cascade.

## Introduction

To meet energy demands during winter, hibernators rely on body fat stores that they have accumulated during the previous summer (Geiser and Kenagy 1993). Fatty acids are mobilized in a coordinated way: during lipolysis, shorter-chain fatty acids and unsaturated fatty acids are released first (Connor et al. 1996; Raclot 2003). Nevertheless, polyunsaturated fatty acids (PUFAs), notably those of the omega-6 series, accumulate in white adipose tissue (WAT) of many hibernators, suggesting selective retention of these PUFAs, instead of metabolization. This selective mobilization of fatty acids may indicate physiological roles of PUFAs alternative to fuel metabolism.

One implication is related to adaptation to low body temperature (T_b_) during torpor. When fed diets containing plant oils that are rich in omega-6 PUFAs, heterotherms exhibit a higher propensity to use torpor, lengthen torpor bout duration, lower minimal T_b_, and thus increase their energy savings (Bruns et al. 2000; Florant et al. 1993; Frank 1992; Geiser and Kenagy 1987; Geiser and Kenagy 1993; Thorp et al. 1994). Heterotherms also seem to prepare tissues for a life at low T_b_ independently of the dietary uptake of PUFAs. For instance, deer mice (*Peromyscus maniculatus*) have been found to increase the amount of omega-6 PUFAs in leg muscle when exposed to short photoperiod (Geiser et al. 2007), and alpine marmots (*Marmota marmota*) transfer omega-6 PUFAs from WAT to heart and liver phospholipids (PLs) at a high rate shortly before hibernation (Arnold et al. 2011). In hibernators, these changes in lipid composition are expected to ensure proper body functions at low T_b_ during torpor, possibly through the maintenance of lipid fluidity (Aloia and Raison 1989; Sinensky 1974; Tiku et al. 1996) and/or the regulation of membrane proteins by specific lipids (see also Arnold et al. 2015 for review; Giroud et al. 2013; Ruf and Arnold 2008).

**Fig. 1:**
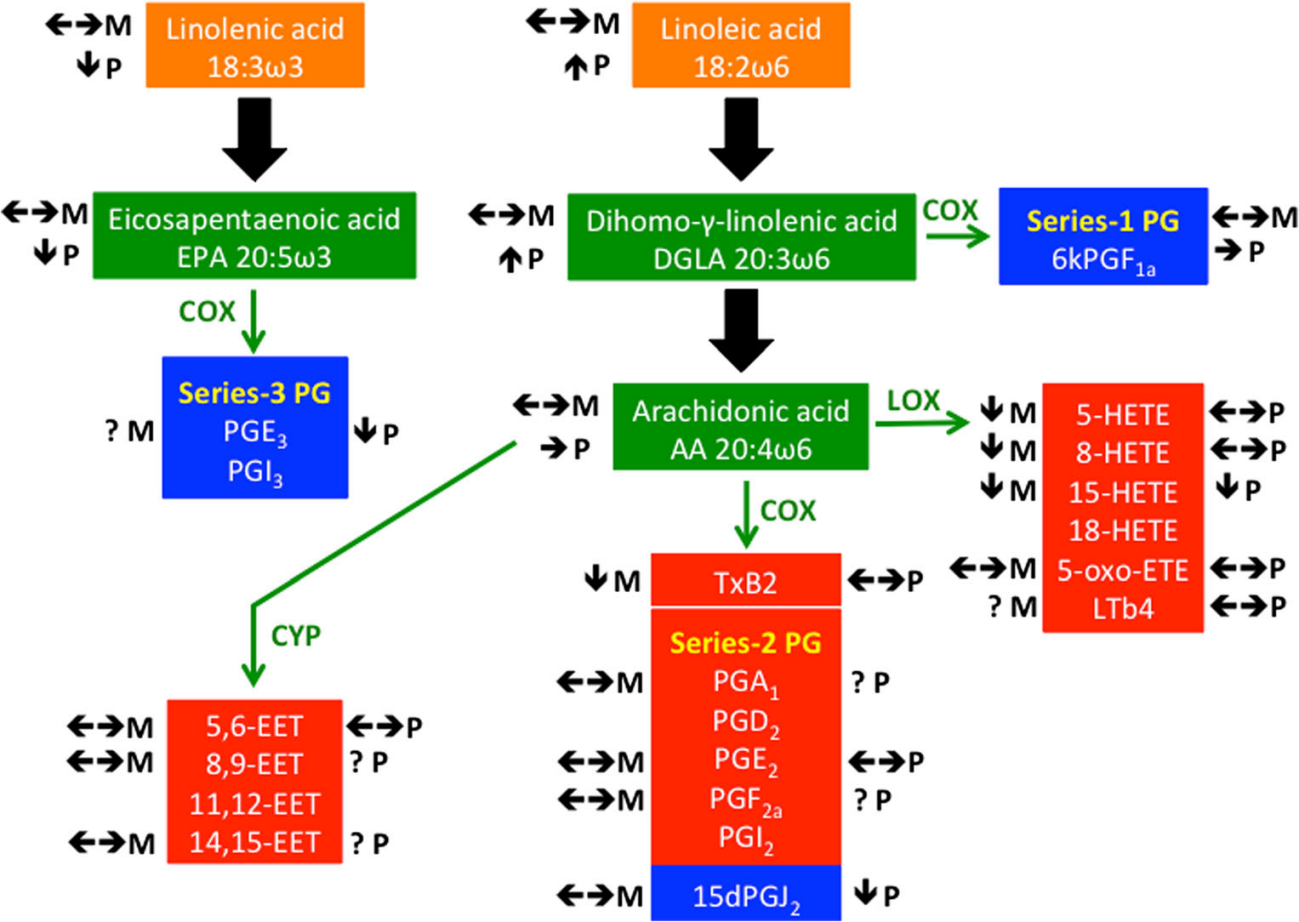
Simplified eicosanoid metabolic pathways from omega-3 and omega-6 fatty acids. The fatty acid precursors (orange), i.e., linolenic acid (18:3 ω3) and linoleic acid (18:2 ω6), are converted into eicosapentaenoic acid (EPA, 20:5ω3) and dihomo-γ-linolenic acid (DGLA, 20:3ω6), respectively. DGLA is further converted into arachidonic acid (AA, 20:4 ω6). The free EPA, DGLA, and AA (green) are then acted upon by the primary metabolic enzymes, i.e., cyclooxygenase (“COX”), lipoxygenase (“LOX”), and cytochrome P450 (“CYP”), and converted to numerous bioactive compounds involved in pro-inflammatory (red) and anti-inflammatory (blue) processes. Directions of changes of the fatty acid precursors and various eicosanoid molecules measured in muscle tissue (“M”) or blood plasma (“P”) of bears in this study are indicated by upward and downward arrows or by horizontal arrows when no significant changes occurred. Question marks (“?”) refer to non-detectable concentrations

Another reason for diverting PUFAs from β-oxidation might be that some omega-6 and omega-3 fatty acids from membrane PL are the precursor pools that serve as substrates for the enzymes of the eicosanoid cascade in most tissues. Typically, eicosanoids derived from omega-6 precursors, such as arachidonic acid (20:4 ω6), exert pro-inflammatory effects, whereas those derived from omega-3 fatty acids have anti-inflammatory properties (Fig. 1) (Schmitz and Ecker 2008). Beyond their roles in inflammatory processes (Levick et al. 2007; Node et al. 1999; Node et al. 2001), eicosanoids also exert complex functions over many other bodily systems, such as thermoregulation (Prendergast et al. 2002; Ruan et al. 2008; Ueno et al. 1982) and the cardiovascular system (Hoebel and Graier 1998; Levick et al. 2007; Rzigalinski et al. 1999). For instance, series-2-prostaglandins that are derived from one of the cyclooxygenase pathways exert contrasting functions on thermoregulation in hibernators. Prostaglandin D_2_ (PGD_2_) elicits hypothermia (Ueno et al. 1982), whereas the infusion of prostaglandin E_2_ (PGE_2_) has been shown to cause arousal from hibernation concomitant with fever in Golden-mantled ground squirrels, *Callospermophilus lateralis* (Prendergast et al. 2002). Although most physiological functions are downregulated during hibernation, hibernators are capable of maintaining the integrity of key organs and important tissues. For instance, cardiovascular function and brain integrity are preserved (Andrews 2007; Johansson 1996; Magariños et al. 2006; von der Ohe et al. 2006; von der Ohe et al. 2007; Wang et al. 2002), loss of muscle mass and strength are minimized (Harlow et al. 2001; Lohuis et al. 2007; Mahlert et al. 2018), and bone structure is maintained (Mahlert et al. 2018; McGee-Lawrence et al. 2015). Given the large influence of eicosanoids, characterizing the seasonal changes of eicosanoid levels in hibernators is of great interest for determining whether eicosanoid metabolism might play a role in regulating these physiological processes.

To date and to our knowledge, only one study has investigated eicosanoid metabolism in relation to hibernation under free-living conditions (Arnold et al. 2012). This is of major importance since laboratory diets fail to reflect natural diet selection of free-living animals that, as reported above, constrain hibernation physiology and phenology. Further, this one study was conducted in alpine and yellow-bellied marmots, which are typical hibernators (of less than 10 kg). Here, we present a unique dataset from a large (more than 10 kg) hibernator, the free-ranging brown bear (***Ursus arctos***), studied in its natural environment. The data are unique since the Scandinavian Brown Bear Research Project, we are part of, is the only team that has the experience of capturing free-living hibernating bears. Although bears hibernate at T_b_ reduced by only few degrees, i.e. from ~ 37 °C in euthermia to ~ 33 °C in torpor (Evans et al. 2016), ursids can still reduce their metabolism during hibernation down to 25% of basal rates (Tøien et al. 2011). In particular, hibernating bears reach minimum specific metabolic rate that lies within the same range of those occurring in small hibernators (Heldmaier et al. 2004; Ruf and Geiser 2015). “This implies that bears use the entire mammalian scope of metabolic inhibition,” i.e., suppression of metabolism, during torpor (Heldmaier 2011). In this study, we aimed at investigating the cascade of eicosanoids in bears during winter hibernation and the summer active period, along with the seasonal changes of omega-3 and omega-6 fatty acid pathways, i.e., lipoxygenase, cytochrome P450, and cyclooxygenase, involved in the eicosanoid cascade.

## Material and methods

### Study area

The study area encompassed about 21,000 km^2^ in south-central Sweden (61°N, 15°E). The topography in this region is rolling hills, with < 10% above 750 m above sea level. The area is forested and dominated by Scots pine (*Pinus sylvestris* L.) and Norway spruce (*Picea abies* H. Karst). The area is heavily used by the forestry industry, with 8% of the land in recent clear-cuts and 40% of the trees under 35 years of age (Moe et al. 2007). The human population is low, but there is an extensive network of forestry roads and some paved roads. The area is heavily used by hunters with dogs, not only during the moose (*Alces alces*) hunting season in September and October but also during the bear hunting season, which begins on 21 August and ends when the quota of 200–300 bears is filled, usually mid- to late September (Swenson et al. 2017). The total population estimate for Sweden was 2968–3667 brown bears in 2008 (Kindberg et al. 2011). This hunting period can overlap with the pre-denning period [usually from early-October to early-December] that is characterized by an accumulation of energy reserves and den site selection, essential for the success of winter hibernation (Evans et al. 2016). Bears enter the den when snow comes and ambient temperature falls down to 0 °C, whereas termination of denning seems to be determined by physiological cues (Evans et al. 2016). In the southern area, denning of male brown bears lasts on average for 161 days (end-October to start-April) and duration of their denning decreases with increasing age and body mass (Manchi and Swenson 2005). Males emerge from dens earlier than females, whose denning period is influenced by their reproductive status, i.e., pregnant females stay the longest time in their dens (Manchi and Swenson 2005). Most den abandonments occurred early in the denning season; a recent study documented that 22% of bears changed dens during winter and only 4% after mid-December (Sahlén et al. 2015).

### Animals and sample collection

Brown bears have been captured annually by the Scandinavian Brown Bear Research Project and fitted with neck collars, which included a global positioning system (GPS), dual-axis motion sensors (to monitor activity), very-high-frequency (VHF) transmitters, and a global system for mobile communication (GSM) modem (Vectronic Arospace GmbH, Berlin, Germany). As a backup to relocate bears if the collar malfunctioned, VHF transmitters were implanted into the abdomen (Telonics, Inc., Mesa, Arizona, USA) (Arnemo and Evans 2017). GPS positions were recorded every 30 min. Bears that were the offspring of marked females were followed from birth; otherwise, age was determined by counting the annuli of a cross-section of the premolar roots (Matson et al. 1993). All captures and subsequent interventions carried out on the animals were approved by the Ethical Committee on Animal Experiments, Uppsala, Sweden (application no. C47/9) and the Swedish Environmental Protection Agency. Further, all experiments were performed in accordance with relevant guidelines and regulations.

Ten bears (3 males, 7 females, 2–4 years old, 21–58 kg) were used for this study. Males and females had similar body mass in summer (*t* = 0.58, *p* = 0.60) as well as during winter (*t* = 0.90, *p* = 0.41). All bears hibernated alone and were captured during winter hibernation in February 2011 and 2012 by darting them in their den, as previously described (Arnemo and Evans 2017; Evans et al. 2012). Once anesthetized, we took each of the bears out of the den (during winter) and placed them on an insulated blanket. The same individuals (22–72 kg) were recaptured, when active (T_b_ ~ 38 °C) in June 2011 and 2012, by darting from a helicopter (Arnemo and Evans 2017; Fahlman et al. 2011). The same samples were taken from these bears during both seasons. Sufficient quantities from the muscle tissue (*vastus lateralis*) biopsies were available from 7 bears in summer and 7 bears in winter, including 4 bears (1 male, 3 females) that were captured and sampled in both seasons. Sufficient amount from blood samples were available from 10 bears in summer and 9 bears in winter, including 9 bears (3 males, 6 females) with paired blood samples. Blood samples were centrifuged at 3500 rpm for 15 min at 5 °C. Plasma and muscle tissue were snap-frozen and stored at − 80 °C for subsequent lipidomics analyses.

### Total FAME analysis

We extracted lipids from 1 mg of muscle and 10 μl of plasma by using a procedure described by Bligh and Dyer (Bligh and Dyer 1959) in dichloromethane/methanol/water (2.5:2.5:2.1, *v*/*v*/*v*), in the presence of the internal standards glyceryl triheptadecanoate (2 μg). Lipid extracts were hydrolyzed in KOH (0.5 M in methanol) at 50 °C for 30 min and transmethylated in boron trifluoride methanol solution 14% (SIGMA, 1 ml) and heptane (1 ml) at 80 °C for 1 h. After adding water (1 ml) to the crude extract, fatty acid methyl esters (FAMEs) were extracted with heptane (3 ml), evaporated to dryness, and dissolved in ethyl acetate (20 μl). FAMEs (1 μl) were analyzed by gas-liquid chromatography (Lillington et al. 1981) on a Clarus 600 Perkin Elmer system using a Famewax RESTEK fused silica capillary columns (30 m × 0.32 mm i.d., 0.25 μm film thickness). Oven temperature was programmed from 110 to 220 °C at a rate of 2 °C per min, and the carrier gas was hydrogen (0.5 bar). The injector and the detector temperatures were set to 225 and 245 °C, respectively.

### Oxylipin quantification

For extraction, each frozen tissue was crushed with a FastPrep ®-24 Instrument (MP Biomedical) in 1 ml of HBSS (Invitrogen). After 2 crush cycles (6.5 m/s, 30 s), 10 μl were withdrawn for protein quantification.

Homogenate (the equivalent of 10 mg of muscle) or 100 μl of plasma were withdrawn for oxylipins analyses, and the final volume was completed to 900 μl with HBSS. Three hundred microliters of cold methanol and 5 μL of internal standard (Deuterium labeled compounds) were added. After centrifugation at 900 g for 15 min at 4 °C, supernatants were transferred into 2 ml 96-well deep plates and diluted in H_2_O to 2 ml. Samples were then submitted to solid-phase extraction (SPE) using a HRX 96-well plate (50 mg/well, Macherey Nagel) pretreated with MeOH (2 ml) and equilibrated with 10% MeOH (2 ml). After sample application, the extraction plate was washed with 10% MeOH (2 ml). After drying under aspiration, lipid mediators were eluted with 2 ml of MeOH. Prior to LC-MS/MS analysis, samples were evaporated under nitrogen gas and reconstituted in 10 μl on MeOH. LC-MS/MS analyses were performed as previously described (Le Faouder et al. 2013). Briefly, lipid mediators were separated on a ZorBAX SB-C18 column (2.1 mm, 50 mm, 1.8 μm) (Agilent Technologies) using Agilent 1290 Infinity HPLC system (Technologies) coupled to an ESI-triple quadruple G6460 mass spectrometer (Agilent Technologies). Data were acquired in multiple reaction monitoring (MRM) mode with optimized conditions (ion optics and collision energy). Peak detection, integration, and quantitative analysis were carried out using Mass Hunter Quantitative analysis software (Agilent Technologies) based on calibration lines built with commercially available eicosanoid standards (Cayman Chemicals).

### Statistical analyses

Lipidomics analyses identified and quantified total fatty acids of the omega-3 (linolenic acid 18:3 ω3 and eicosapentaenoic acid 20:5 ω3) and omega-6 (linoleic acid 18:2 ω6, dihomo-γ-linolenic acid 20:3 ω6, arachidonic acid 20:4 ω6) families, as well as free eicosanoids with pro-inflammatory agents, i.e., epoxyeicosatrienoic acids (“5,6-EET,” “8,9-EET,” “14,15-EET”), leukotriene b4 (“LTb4”), hydroxyeicosatetraenoic acid (“5-15-8-HETE”), thromboxane B2 (“TxB2”), prostaglandins (“PGA_1_,” “PGE_2_,” “PGF_2a_”), and anti-inflammatory actions, i.e., prostaglandin E_3_ (“PGE_3_”), 15-deoxy-d-12, 14-prostagladin J2 (“15d-PGJ_2_”), and 6 keto prostaglandin F1a (“6kPGF_1a_”). Figure 1 provides further details concerning metabolic pathways of eicosanoids derived from the omega-3 and omega-6 fatty acids.

Data analyses were carried out using SAS 9.4 (SAS Institute, Inc., Cary, North Carolina). Standardized residuals from statistical models were tested for normality using Kolmogorov-Smirnov tests. We used linear mixed-effects models (LMMs) accounting for repeated measurements among animals to test for the effect of season (fixed variable) on the different omega-3 and omega-6 free fatty acids, and on eicosanoids (predicted variable). Analyses were performed using (1) all available samples and (2) only paired samples (9 for plasma and 4 for muscle). As the analysis with paired samples was more conservative, only results of the second analysis (2) are presented. Values are means ± SE.

## Results

### Omega-3 and omega-6 fatty acids

**Fig. 2:**
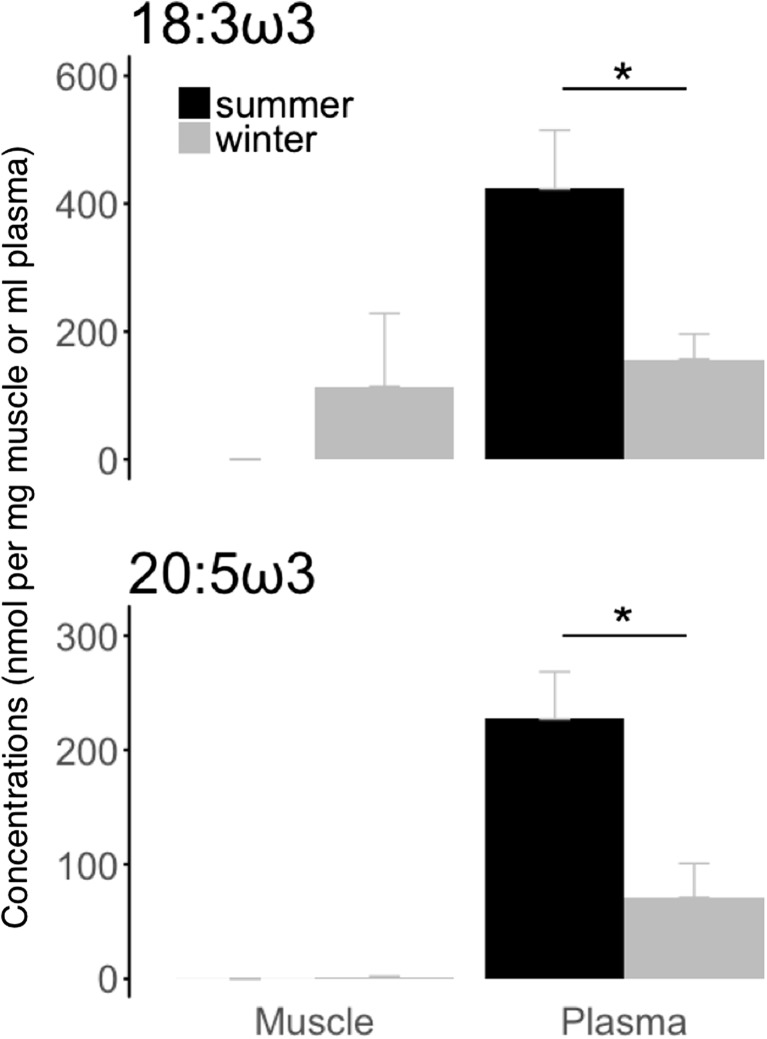
Summer and winter levels of specific omega-3 fatty acids involved in the eicosanoid metabolism. Levels of α-linolenic acid (“18:3 ω3”) and eicosapentaenoic acid (“20:5 ω3”) were assessed in muscle tissue (“muscle”) and blood plasma (“plasma”) from winter-hibernating (“winter”) and summer-active (“summer”) brown bears. Fatty acid levels are means ± SE. Significant differences between winter and summer levels are denoted by an asterisk (*p < 0.05)

**Fig. 3:**
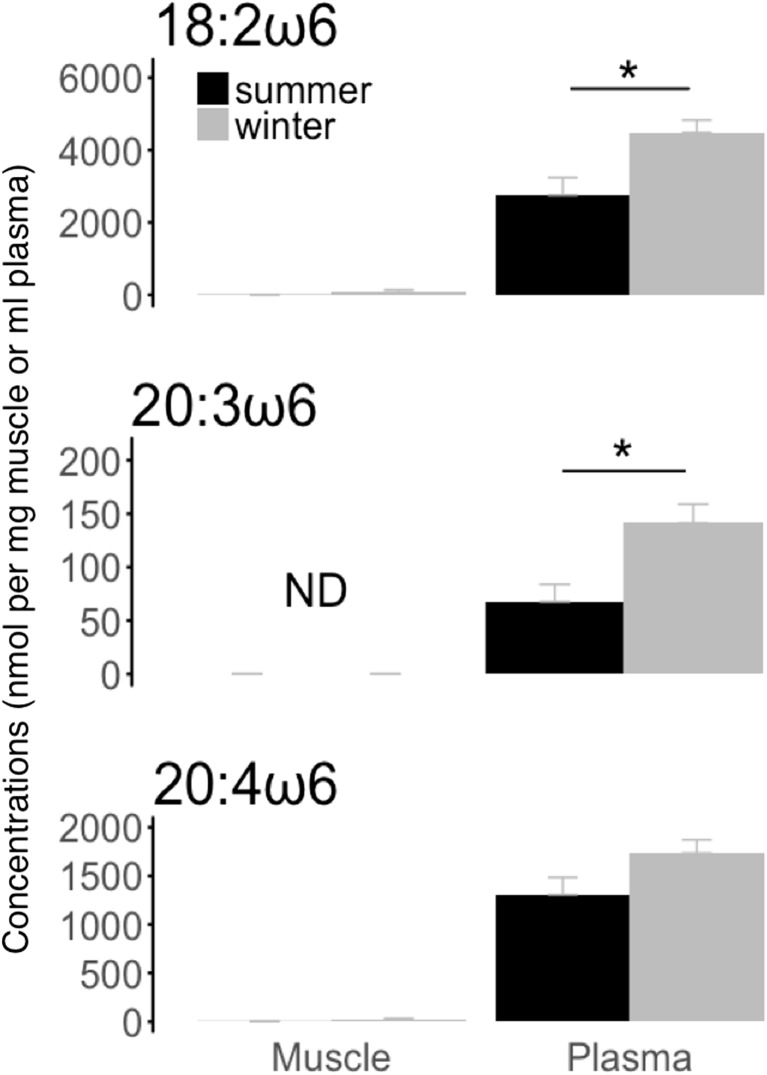
Summer and winter levels of specific omega-6 fatty acids involved in the eicosanoid metabolism. Levels of α-linoleic acid (‘18:2 ω6’), dihomo-γ-linolenic acid (‘20:3 ω6’), and arachidonic acid (‘20:4 ω6’) were assessed in muscle tissue (muscle) and blood plasma (plasma) from winter-hibernating (winter) and summer-active (summer) brown bears. Fatty acid levels are means ± SE. Significant differences between winter and summer levels are denoted by an asterisk (*p < 0.05). “ND” refers to non-detectable concentrations

We found significantly lower plasma levels of linolenic acid (18:3 ω3) and eicosapentaenoic acid (20:5 ω3) in hibernating bears during winter compared to summer active animals (Fig. 2). Conversely, levels of linoleic acid (18:2 ω6), dihomo-γ-linolenic acid (20:3 ω6), but not arachidonic acid (20:4 ω6), were significantly higher in blood plasma of bears in winter hibernation than during the summer active season (Fig. 3). No significant winter-summer differences were detected in muscle tissue for any of those fatty acids (Figs. 2 and 3).

### Pro-inflammatory eicosanoids

**Fig. 4:**
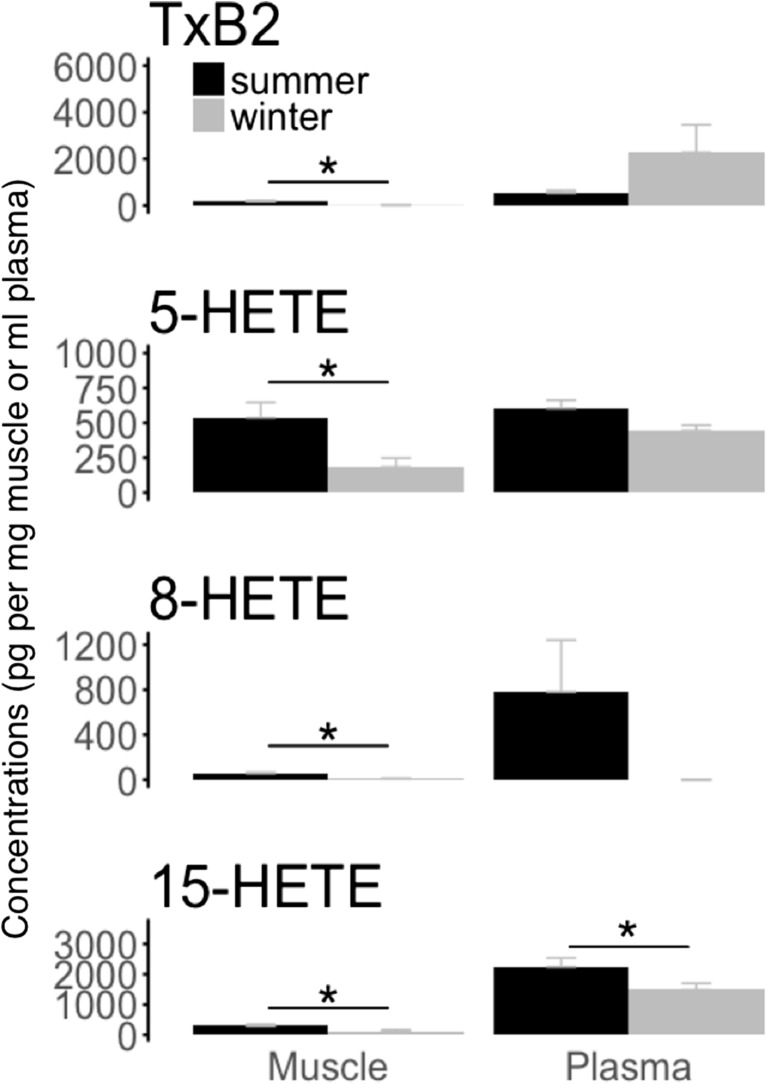
Summer and winter levels of eicosanoids with pro-inflammatory effects. Levels of thromboxane B2 (“TxB2”) and 5-, 15-, and 8-hydroxyeicosatetraenoic acids (“5-HETE’,” “15-HETE,” “8-HETE”) were measured in muscle tissue (muscle) and blood plasma (plasma) from winter-hibernating (winter) and summer-active (summer) brown bears. Eicosanoid levels are means ± SE. Significant differences between winter and summer levels are denoted by an asterisk (*p < 0.05)

We found lower level of TxB2 in muscle tissue of hibernating bears compared to the summer active animals, whereas TxB2 plasma levels remained unchanged between seasons (Fig. 4). Further, levels of 5-HETE, 8-HETE, and 15-HETE were significantly lower in muscle tissues of bears in winter hibernation than during the summer active period (Fig. 4). Also, bears showed lower plasma levels of 15-HETE, but not of 5-HETE and 8-HETE, in winter hibernation than during the summer (Fig. 4). Both plasma and muscle levels of 5,6 EET, as well as muscle concentration of 8,9 EET, showed non-significant tendencies to be lower in hibernating bears than in active animals during summer (Table 1). However, we found no significant seasonal changes in other pro-inflammatory eicosanoids, such as 5-oxo-ETE, LTb4, PGA_1_, PGE_2_, PGF_2a_, and 14,15 EET in muscle tissue and blood plasma of bears (Table 1).

**Table 1:** Summer and winter levels of eicosanoids with pro-inflammatory and anti-inflammatory effects in winter-hibernating and summer-active brown bears. Eicosanoid concentrations are means ± standard errors and correspond to pg mg^−1^ of muscle tissue or pg ml^−1^ of blood plasma. ND refers to non-detectable concentrations

Tissues	Effect	Variables	Concentrations		*P* values
			Summer	Winter	
Muscle					
	Pro-inflammatory				
		5-oxo-ETE	4300.01 ± 1559.18	2660.50 ± 1275.52	0.171
		LTb4	ND	ND	ND
		PGA_1_	6.31 ± 3.54	0.01 ± 0.01	0.233
		PGE_2_	9.70 ± 2.51	6.13 ± 1.83	0.502
		PGF_2a_	17.39 ± 5.38	8.35 ± 2.19	0.345
		5,6 EET	117.95 ± 32.48	42.63 ± 19.40	0.093
		8,9 EET	522.58 ± 112.08	195.99 ± 47.59	0.079
		14,15 EET	126.79 ± 53.17	36.99 ± 27.97	0.110
	Anti-inflammatory				
		6kPGF_1a_	61.91 ± 15.80	42.30 ± 7.85	0.925
Plasma					
	Pro-inflammatory				
		5-oxo-ETE	4790.60 ± 1098.86	10,027.09 ± 2952.30	0.230
		LTb4	169.22 ± 13.58	153.81 ± 8.27	0.216
		PGA_1_	ND	ND	ND
		PGE_2_	111.85 ± 29.11	100.04 ± 22.24	0.984
		PGF_2a_	ND	ND	ND
		5,6 EET	566.36 ± 111.89	407.02 ± 81.23	0.093
		8,9 EET	ND	ND	ND
		14,15 EET	ND	ND	ND
	Anti-inflammatory				
		6kPGF_1a_	363.66 ± 138.24	374.17 ± 39.31	0.930

### Anti-inflammatory eicosanoids

**Fig. 5:**
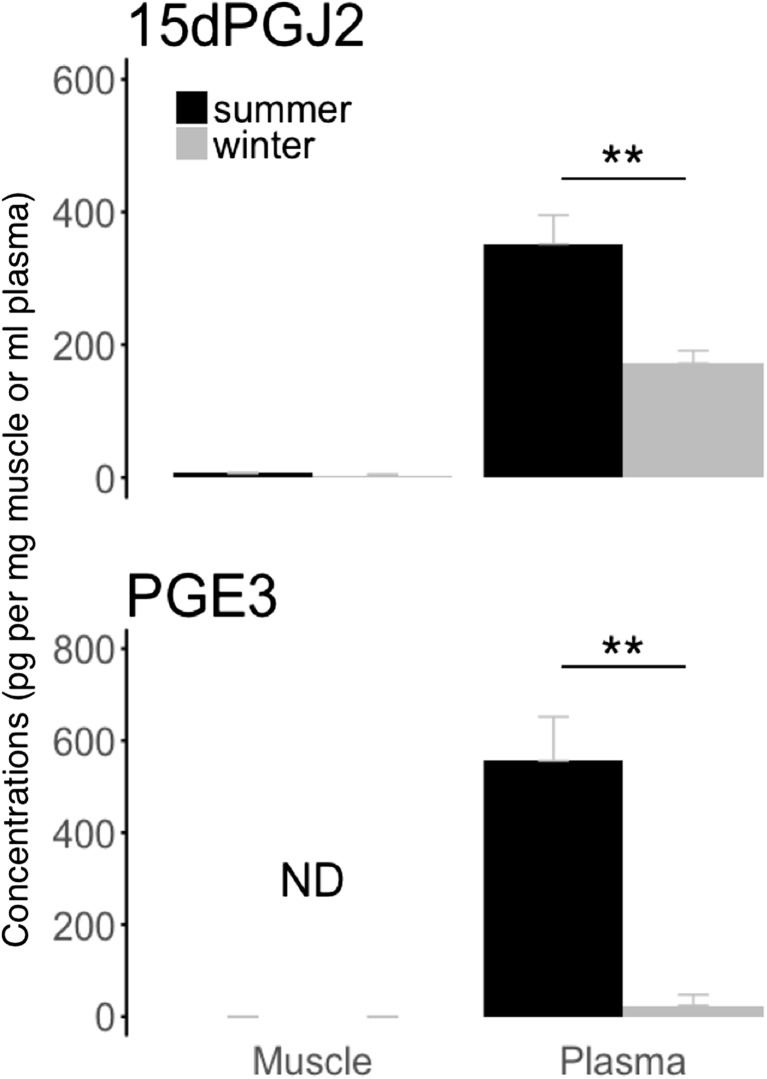
Summer and winter levels of eicosanoids with anti-inflammatory effects. Levels of 15-prostagladin J2 (“15dPGJ2”) and prostaglandin E3 (“PGE3”) were measured in muscle tissue (muscle) and blood plasma (plasma) from winter-hibernating (winter) and summer-active (summer) brown bears. Eicosanoid levels are means ± SE. Significant differences between winter and summer levels are denoted by an asterisk (**p < 0.01). ND refers to non-detectable concentrations

Levels of 15dPGJ_2_ and PGE_3_ were either unchanged or non-detectable in muscle tissue (Fig. 5). Plasma levels of 15dPGJ_2_ and PGE_3_ were significantly lower in winter-hibernating bears compared to summer-active animals (Fig. 5). We found no significant seasonal differences in 6kPGF_1a_ in muscle and blood plasma of bears (Table 1).

## Discussion

In this study, concentrations of the eicosanoids derived from all three pathways were significantly reduced, or remained unchanged, in blood plasma and muscle tissue of free-living bears during winter hibernation compared to the summer active season. Further, those changes were independent of the pro- or anti-inflammatory properties of the eicosanoids. We also observed significant seasonal changes, although of limited amplitude, in specific omega-3 and omega-6 fatty acids involved in eicosanoid metabolism.

Previous studies on hibernators have reported seasonal changes in levels of some prostaglandins, such as PGD_2_ and PGE_2_, in the brain of alpine marmots (Arnold et al. 2012) and Asian Chipmunk, *Eutamias sibiricus* (Takahata et al. 1996). Specifically, PGD_2_ concentration increases during winter when animals lower T_b_ while entering hibernation, and PGE_2_ levels are higher during the summer active season when T_b_ is elevated compared to winter. In the present study, however, we did not find seasonal alterations of any of these eicosanoids in bears that, in contrast to deep hibernators, reduce their T_b_ by only few degrees during winter hibernation, which constitutes the main specificity of the bear hibernation phenotype. This might therefore explain the lack of significant changes in levels of these prostaglandins, the implications of which for hibernation clearly need further studies. Instead, in bears during hibernation compared to summer, we found significant lower plasma levels of other prostaglandins, i.e., 15dPGJ_2_ (a dehydration metabolite of PGD_2_) and PGE_3_, and a reduced muscle concentration of thromboxane (TxB2), all of which are known for their regulatory role in inflammation (see Fig. 1 for pro- or anti-inflammatory roles). Interestingly, seasonal variations of anti-inflammatory non-eicosanoid molecules, such as haptoglobin, were reported in European brown bears, with plasma levels being highest during hibernation compared to other times of the year (Mominoki et al. 2005). This supports the hypothesis that inflammation is an important and central process regulated by several actors during winter hibernation. Among eicosanoids, 15dPGJ_2_ activates both PPARα and γ (Kliewer et al. 1997; Krey et al. 1997; Li et al. 2005), which in turn inhibit nuclear factor κB and thus several inflammatory processes (Poynter and Daynes 1998; Ricote et al. 1999). Furthermore, the series-3 prostaglandins, PGE_3_ and PGI_3_, both of which are derived from eicosapentaenoic acid (20:5 ω3), have anti-arrhythmic effects and counteract the activating influences of PGI_2_ and PGE_2_ on cardiac function (Li et al. 1997). Also, TxB2, produced from arachidonic acid (20:4ω6), is a potent vasoconstrictor and platelet activator. Eicosapentaenoic acid-derived prostaglandins, such as PGE_3_, have been shown to inhibit TxB2-mediated platelet aggregation and promote vasodilatation (Weber et al. 1986). During months of fasting and immobilization, hibernating bears are protected from thrombotic complications and muscle wasting (for review, see Stenvinkel et al. 2018). Such phenomena are also known to occur in small hibernators (de Vrij et al. 2014; Mahlert et al. 2018), although their patterns of eicosanoids change differ from the one of the bears in this study. The understanding of such phenomena therefore clearly deserves further studies. In respect to hibernating bears, animals tolerate extended periods of low heart rate without developing thromboembolic events or cardiac dilatation. The protection against vascular disease may be due to changes in the coagulation pathways, which are under the regulation of oxylipins such as prostaglandins (for review, see Caligiuri et al. 2017). Also, black bears are able to retain muscle integrity and to completely spare their muscle cell number or size and strength throughout winter dormancy (Harlow et al. 2001; Lohuis et al. 2007). In our study, levels of prostaglandins in muscle were not reduced, as those of other eicosanoids, but instead unchanged in bears during winter compared to summer. Maintaining levels of prostaglandins in muscle during winter similar to those in summer can likely contribute to the mechanisms of muscle sparing in bears during hibernation. Indeed, supplementation with arachidonic acid (20:4 ω6) leads to increased size and protein content of C2C12 myotubes, an effect mediated by enhanced cyclooxygenase activity and prostaglandin synthesis, leading specifically to augmented secretion of PGF_2a_ and PGE_2_ (Markworth and Cameron-Smith 2012). Therefore, the results of this study suggest that reduced levels of some eicosanoids ensure the functioning of the heart and cardiovascular system in hibernating bears and that maintaining relatively high levels of prostaglandins in winter contributes to the maintenance of the muscle integrity of bears during hibernation.

In the present study, we also found significant alterations of eicosanoids derived from the lipoxygenase and cytochrome P450 pathways. Muscle concentrations of (5-, 8-, 15-) HETEs and plasma levels of 15-HETE were significantly lower in bears in winter hibernation compared to the summer active period. Furthermore, 5,6-EET levels in plasma and muscle, although not statistically significant, tended to be lower in hibernating bears than in active animals during summer. HETEs are known to act on gene expression through the regulation of PPARs. For instance, 8-HETE interacts preferentially with the α-isoform of PPARs [PPARα] (Kliewer et al. 1997), which are key players in the much larger picture of energy homeostasis, in lipid metabolism, in adipogenesis, in cell cycle regulation, and in the inflammatory responses (Kliewer and Willson 1998; Latruffe and Vamecq 1997; Schoonjans et al. 1996). The eicosanoid EETs, which also are derived from arachidonic acid, have been shown to have effects on cardiomyocyte function. For instance, 8,9-EET inhibits cardiac Na^+^ channels and produces a hyperpolarization shift in the steady-state membrane potential (Lee et al. 1999). Also, 11,12-EET can have direct inhibitory effects on cardiac L-type Ca2+ channels reconstituted into planar lipid bilayers (Chen et al. 1999). Another study (Xiao et al. 2004), however, reported the opposite effect of 11,12-EET that accelerated Ca^2+^ current, through increased cAMP-dependent phosphorylation of Ca^2+^ channels, when applied to a cardiac ventricular preparation. Taken together, these studies suggest that EETs can positively or negatively modulate the activity of Ca^2+^ channels depending on the cellular energy requirements. Given the fact that the activity level of ion channels is one of the main determinants of the resting metabolic rate of living organisms (Rolfe and Brown 1997; Smith et al. 2013), the inhibitory effects of EETs might contribute to the reduction of metabolic rate that occurs in preparation and during hibernation. Similarly, effects of EETs on specific ion [Na^+^] channels can contribute to the stabilization of the cardiac potential, hence to the reduction of heart rate variability of the animals while entering into torpor. In brown bears, it has been recently reported that heart rate variability, a proxy of sympathetic nervous system activity, drops dramatically once the bear enters the den (Evans et al. 2016), suggesting the occurrence of metabolic suppression linked with denning in bears. Hibernators rely on both a temperature effect, i.e., Arrhenius effect, and metabolic suppression to reduce their metabolic rate during hibernation (Geiser 2004). Large hibernators, such as bears, rely to a larger extent on active metabolic suppression than passive body cooling to achieve depressed metabolism during hibernation (Heldmaier 2011; Tøien et al. 2011). Hence, EET eicosanoids might likely be involved in regulating heart rate and function at low metabolic level during hibernation in bears.

## Conclusion

In this unique study on free-living hibernating bears, we observed significant seasonal changes in the omega-3 and omega-6 pathways at the origin of the eicosanoid cascade. Concentrations of the products of the lipoxygenase, cytochrome P450, and cyclooxygenase pathways decreased significantly, or remained unchanged, in blood plasma and muscle tissue of bears during winter hibernation compared to the summer active period. These changes were independent of the pro- or anti-inflammatory properties of the eicosanoids. Taken together, these findings suggest that hibernation in a large mammal is associated with a depressed state of the eicosanoid cascade. Whether this plays a role in the various sparing abilities of hibernating bears or simply reflects the hypometabolic state associated with hibernation remains to be determined.
